# Grain size and grain depth restrict oxygen movement in leaky hermetic containers and contribute to protective effect

**DOI:** 10.1016/j.jspr.2016.06.006

**Published:** 2016-10

**Authors:** Scott B. Williams, Larry L. Murdock, Kabita Kharel, Dieudonne Baributsa

**Affiliations:** Department of Entomology, Purdue University, 901 W. State Street, West Lafayette, IN 47907, USA

**Keywords:** *Callosobruchus maculatus*, Stored grain, Seed damage, Gas diffusion, Hermetic storage

## Abstract

Postharvest insect pests threaten the nutritional and financial security of smallholder farmers in the developing world. Hermetic storage, a technology that protects grain against insects by blocking their supply of oxygen, alleviates the problem of insect-caused losses. PICS (Purdue Improved Crop Storage) bags represent one hermetic technology that improves food availability and incomes of farmers. The polyethylene liners of PICS bags are sometime damaged during use, acquiring small holes or tears. Observations in the laboratory and field suggest that insect development remains localized around the point where the bag is damaged. We hypothesized that the grain within a hermetic container that has minimal localized damage (such as an insect hole), helps retard leakage of oxygen into the bag and contributes to limiting insect damage and to the overall protective effect. To test this hypothesis, we filled 4 cm dia. by 10 cm long PVC pipes with *Callosobruchus maculatus* (F.) infested cowpeas and sealed them with caps having a single, insect-sized hole in its center. A vertical tube positioned above the cowpea-filled PVC pipe was filled with one of three different grains (sesame, sorghum, and maize) to different depths (0, 5, 15, 30, 50 cm). Seed size and grain barrier depth significantly reduced the level of bruchid damage to the stored cowpea in the PVC container. Smaller sized grains used for the barriers retarded insect development more effectively than larger sized grains, while deeper grain depth was more effective than shallower barriers. The grain held in a hermetic container contributes in a small, but significant, way to the effectiveness of the containers.

## Highlights

• Grain may reduce airflow into damaged hermetic container and reduce insect damage to seed.
• Infested cowpea was buried to different grain depths to determine its effects on damage severity.
• Greater depth resulted in slower population growth and less seed damage.
• Smaller seed size reduced adult development and larval activity.

## 1 Introduction

Insect pests that damage grain during postharvest storage are a threat to food security, especially in the developing world. There, postharvest losses to insects can reduce food availability by 20–50% (Keil, 1988, Pantenius, 1988, Boxall, 2002, Mulungu et al., 2007). Lack of access to reliable and affordable pest control methods force many smallholder farmers to sell their grain at harvest when the price is at the low point of the year and buy it back later when food needs demand they purchase it and prices are higher (Boxall, 2002, Jones et al., 2011, Njoroge et al., 2014).

Hermetic storage containers (metal silos, drums, PICS and GrainPro bags, etc.) address the problem of small-scale grain storage (Moussa et al., 2014). Sealed hermetic containers prevent the flow of oxygen from outside into the grain. Any insects present in the grain when it is placed in the hermetic container use up the limited oxygen and create conditions that are unsustainable for them (Oxley and Wickenden, 1963, Quezada et al., 2006, Murdock et al., 2012).

The adoption of PICS bags in Africa has grown steadily since 2007 with 7 million bags having been purchased thus far. Fifty percent of the cowpea not sold at harvest is now stored in these flexible containers or in other types of hermetic containers (Baributsa, 2014, Moussa et al., 2014, Ibro et al., 2014). Farmers who use these bags have enjoyed lower rates of pest damage, higher grain quality, and improved selling prices at the market (Baributsa et al., 2010).

Farmers are encouraged by PICS’ promise of better grain storage, as evidenced by the continued sales of the bags (Murdock and Bauoa, 2014). Even so, some farmers have expressed concerns about grain stored in bags that may have small leaks. Handling the bags increases the likelihood of mechanical damage and certain insect species (e.g. *Callosobruchus maculatu*s and *Prostephanus truncatus*) can chew holes through the liners. This will permit airflow into the bags and raises the possibility of subsequent insect damage to the grain (Baoua et al., 2014, Hell et al., 2014). However, several years of observation in the field indicate that damage to the grain in such bags is minimal (Baoua et al., 2014). In short, despite localized breaks in the airtight seal of the bag, PICS bags continue to be effective in preventing postharvest losses.

Understanding why small holes or tears in the polyethylene liners do not result in failure of the bags’ ability to protect grain may provide insight that could lead to making the bags more effective. Here, we hypothesize that the grain itself contributes to the PICS bags’ protective action. It is well-known that grain bulk can contribute to the resistance to diffusion of gases through the storage environment (Shunmugam et al., 2005, Haung et al., 2013) and that different grains facilitate different rates of diffusion (Singh et al., 1984). Accordingly, we investigated the role grain may serve in the protective performance of hermetic containers compromised by the presence of small holes or tears.

## 2 Methods

### 2.1 Infested grain preparation

Cowpea bruchids (*Callosobruchus maculatu*s) were obtained from laboratory colonies maintained on cowpea. Black-eye cowpea, variety #8046 (Wax Co., Armory, MS USA) was used for all trials. The grain was held in a freezer at 0 °C for 5 days prior to each trial to ensure it contained no living insects. Four days before setting up each experiment, 2 L of cowpea were removed from the freezer and divided between two glass jars. One jar was heavily infested with *C. maculatus* adults from the laboratory colony. The second jar, with no insects present, was sealed and returned to the freezer.

One day before each trial, the sealed jar was removed from the freezer and given time to warm to room temperature. The adult bruchids in the first jar were removed by sifting using a No. 18 sieve. The two quantities of grain, infested and uninfested, were then mixed together in a 17 L bucket to create a 2 L, 50:50 mixture of infested and uninfested cowpea. Four samples of 100 seeds were removed from the mixture and examined under a magnifying lens. The mean number of infested cowpeas-those cowpeas possessing at least one bruchid egg on its surface-out of each sample of 100 was recorded (Table 1).

### 2.2 Experimental setup

**Fig. 1:**
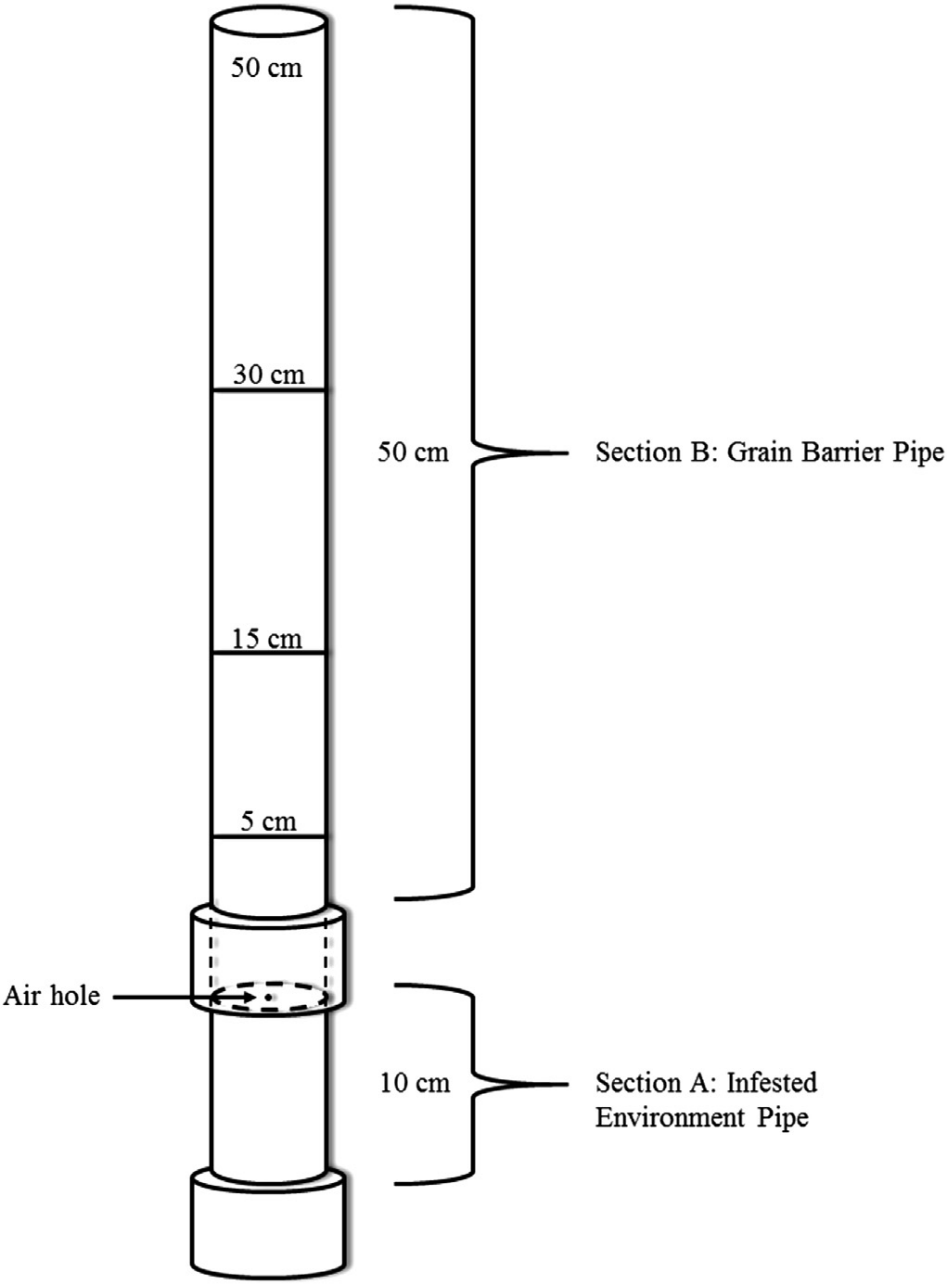
Schematic of grain barrier pipe system. The 10 cm, Section A pipe was filled with 100 mL of infested cowpea and then sealed with a cap. The cap contained a single inlet hole (1.5 mm diameter) for permitting airflow. The 50 cm, Section B pipe was filled to different depths (0, 5, 15, 30 and 50 cm) with one of our three barrier grains (maize, sorghum, or sesame).

**Fig. 2:**
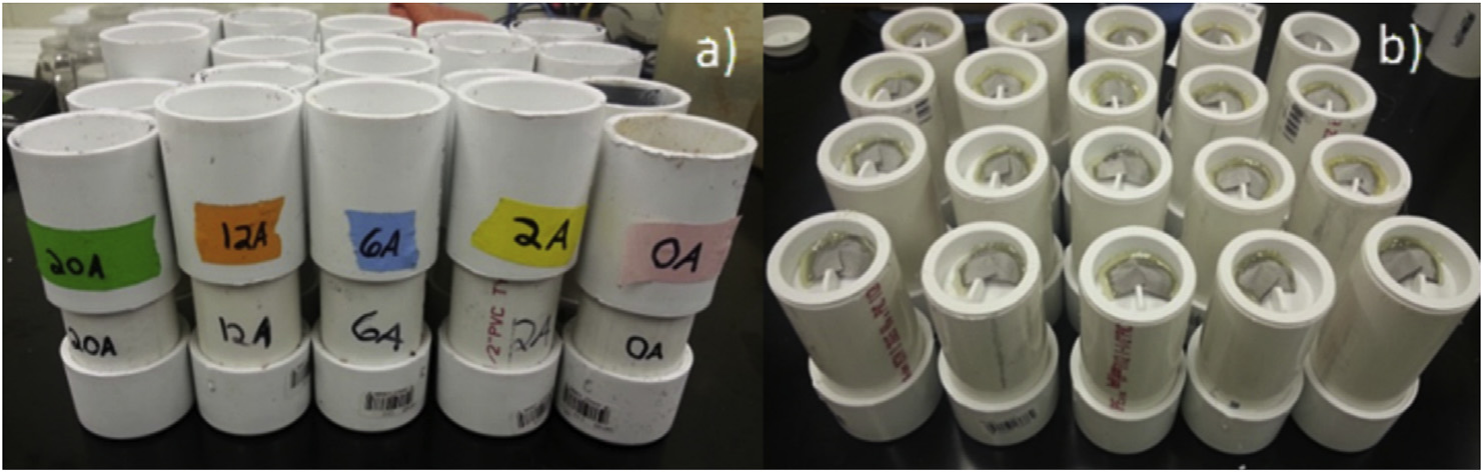
Experimental hermetic containers used for the grain barrier study (a). Stainless steel mesh (b) served as a protective barrier to prevent incidental sealing of the 1.5 mm inlet hole in the containers’ caps.

We used experimental containers constructed from 4 cm diameter PVC pipe (Fig. 1). Each unit was divided into two sections: The first (Section A) was 10 cm long and filled with 100 mL of infested cowpea (Fig. 2a). This section was sealed with a cap using vacuum grease. At the center of the cap was a 1.5 mm inlet hole that served as the only entry point for air into the pipe. A small, rectangular piece of 100 μm, steel mesh was placed over the inlet hole to prevent it from being blocked by grain in the pipe above it (Fig. 2b).

The second section of the experimental container (Section B) was 50 cm long and connected to Section A with a PVC coupling unit. Section B pipes were filled with one of three barrier grains depending on the trial (Trial 1- Sesame, Trial 2- Sorghum, Trial 3- Maize). We selected these grains as our barriers due to the clear differences in the average volume of individual kernels. This gave us the opportunity to determine if kernel size influenced the effectiveness of the grain barrier. Seed volumes (Table 2) are estimates based on published measurements of seed dimensions. The amount of grain used to fill these pipes depended on the grain depth we wished to simulate. Greater depths would result in greater separation of the infested cowpea in Section A from the outside air. Treatment depths ranged from 0 cm for controls to 50 cm for the deepest grain group.

**Fig. 3:**
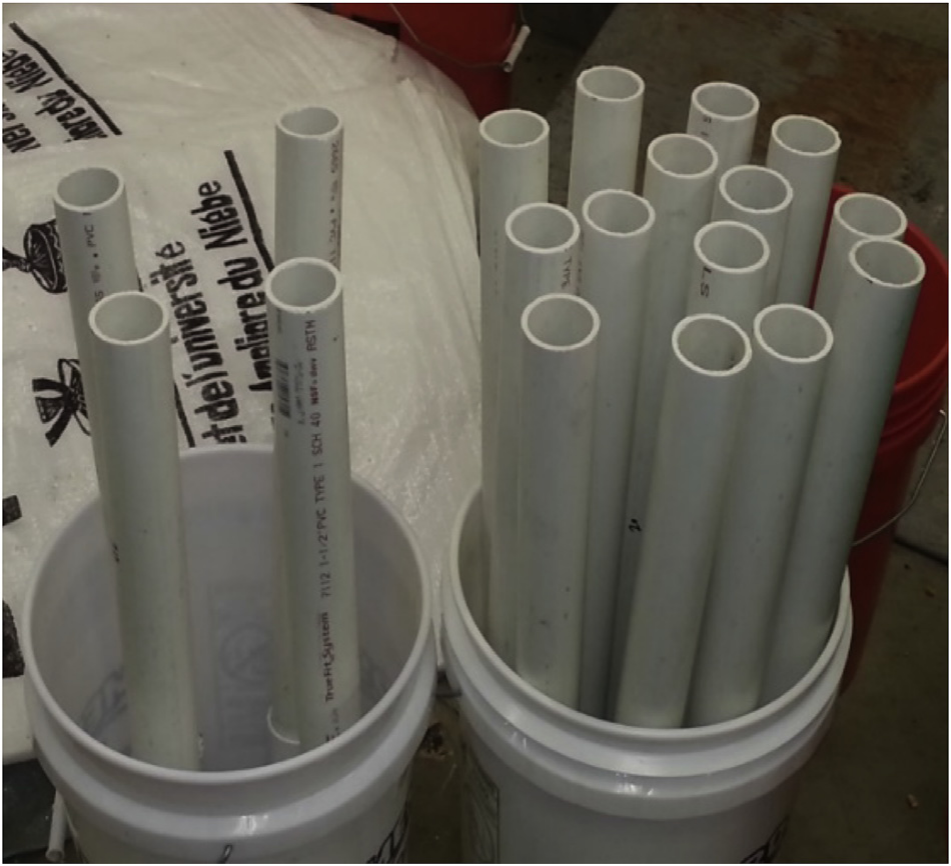
For trials involving maize, sorghum, and sesame, experimental pipes were oriented vertically and stored in a Conviron™ environmental chamber.

All trials lasted 72 d. This period was sufficient for two, full reproductive cycles of the cowpea bruchid. Trials were held inside a Conviron™ environmental chamber in the Purdue Improved Crop Storage (PICS) lab (Fig. 3). Ambient conditions were maintained at 26° C and 30% RH. At the end of the 72 d period, the Section A pipes containing the infested cowpea were placed in a freezer for two weeks. Samples were later removed from each pipe and evaluated.

### 2.3 Evaluation

#### 2.3.1 Seed damage

**Fig. 4:**
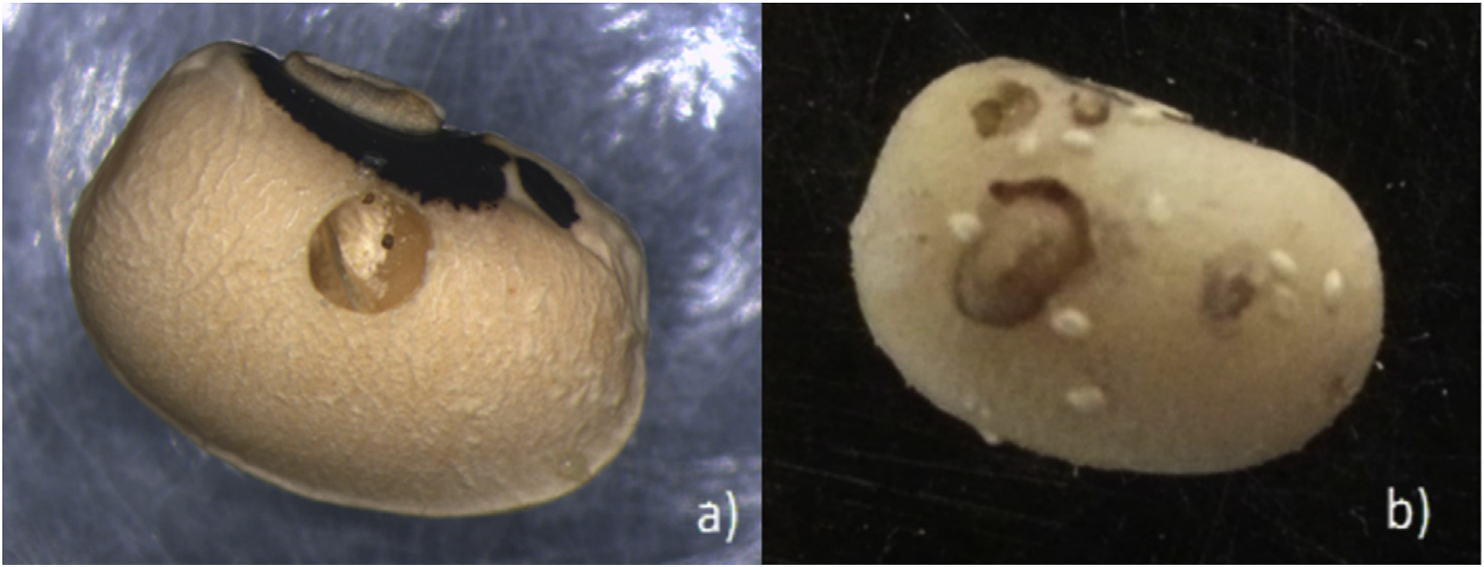
Bruchid (a) Adult Emergence Holes (AEH) are distinguished by their large size relative to the size of the cowpea and regular, round shape. They are formed as the adult beetle leaves the seed. The (b) Larval Air Holes (LAH) are smaller in size and less regular in shape. These holes are formed as a result of stress caused by a decrease in the available oxygen.

Two samples of 100 cowpeas each were removed from the 10 cm pipes and evaluated for insect damage. We recorded three values for each sample: 1) the number of adult emergence holes (AEH) per 100 cowpeas, 2) the number of larval air holes (LAH) per 100 cowpeas, and 3) the dry mass of each sample of 100 cowpeas. An adult emergence hole, which has a regular, circular shape, shows that an adult bruchid has emerged (Fig. 4a). Larval air holes are made by bruchid larvae and are commonly seen when larvae are exposed to low-oxygen environments (Murdock et al., 2012). These holes are smaller than AEHs and are irregularly-shaped (Fig. 4b). Each type of hole was counted via visual inspection of each cowpea from a particular sample.

After counting, each sample was placed in a drying oven at 60° C for 5 d to remove free moisture. Cowpea samples were removed from the oven, allowed to cool, and weighed on an electronic balance. The mass of each sample was recorded to the nearest hundredth of a gram.

#### 2.3.2 Data analysis

The number of AEH and LAH in each cowpea sample was divided by the initial number of infested seeds recorded at the beginning of each trial. For example, if the infestation rate was 50 cowpeas out of 100 at the start of the trial and the number of adult emergence holes per 100 cowpea sample was 103, this resulted in an index value of 2.07. This index value served to standardize observations across our trials and to estimate the relative increases in population size or damage in relation to the initial value.

The normal distribution of assessment values (AEH, LAH, and 100 seed mass) was verified using the Anderson-Darling test. Two-way ANOVA was used to determine the effect of grain depth and grain volume on all three assessment values.

## 3 Results

### 3.1 Effects of grain type

**Fig. 5:**
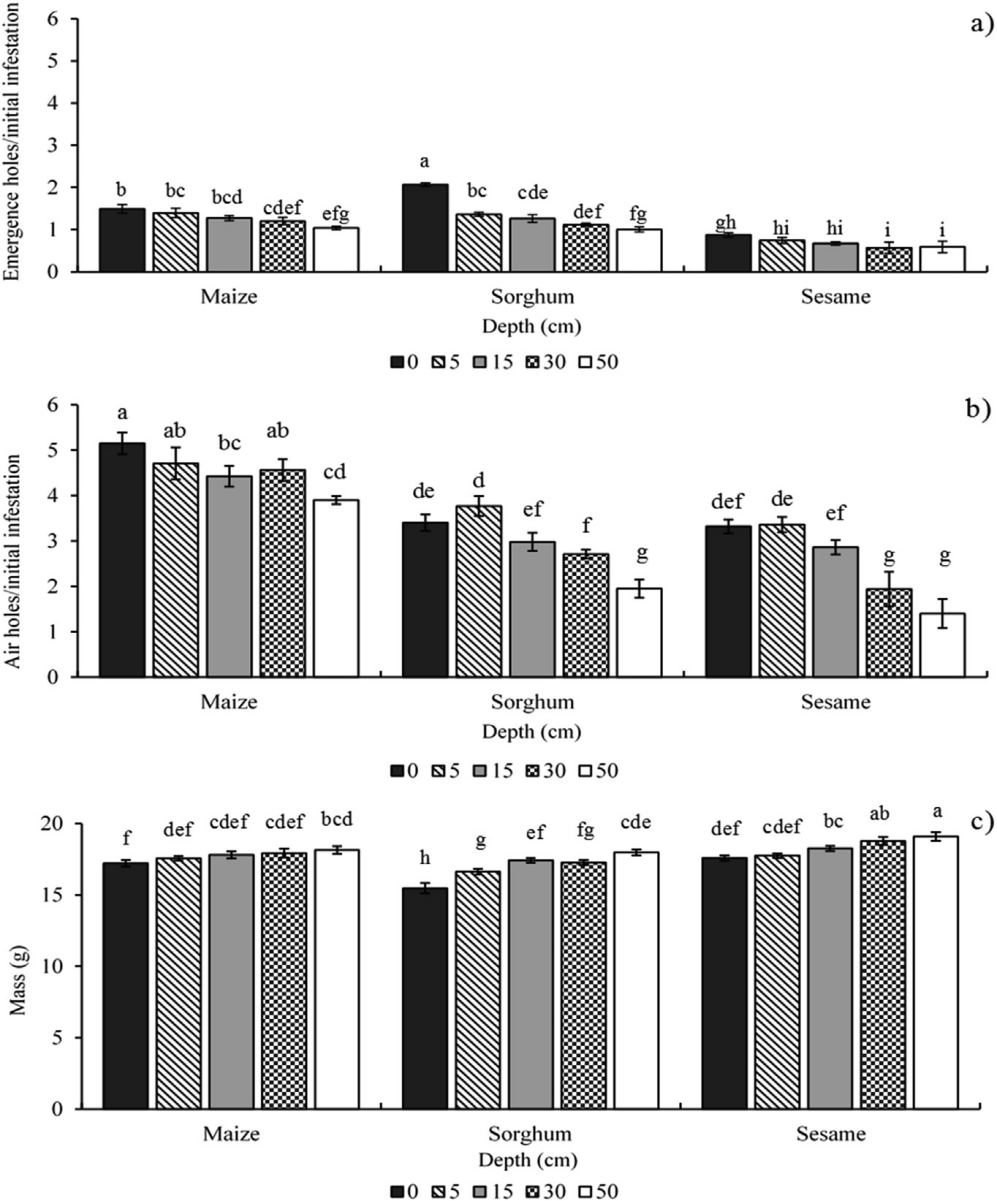
Effect of barrier depth and type of grain used on the severity of damage recorded from samples of infested cowpea. Bars that share letters were not statistically different in value at the α = 0.05 level. The number of emergence holes (a) and air holes (b) relative to the initial infestation rates were significantly influenced by both the volume of grain covering the inlet hole to the 10 cm pipes and the kernel size of the grain. The mass of each 100 seed sample (c) was also negatively affected by the volume of grain and the size of the individual kernels.

Grain depth and seed size had significant, negative effects on the number of AEH and LAH in each cowpea sample (Fig. 5a,b; Table 3). For control pipes, the index value for the AEH relative to the initial infestation ranged between 0.87 (sesame) and 2.07 (sorghum) per 100 seeds. The index value for LAH relative to the initial infestation ranged between 3.32 (sesame) and 5.15 (maize) per 100 seeds. AEH numbers were lower because an adult bruchid only makes one emergence hole as it leaves the cowpea. A larval bruchid can potentially make several air holes over its development. Alternatively, two or more larvae may make a LAH each, but one or both may die and thus not create any emergence holes. This larval mortality then increases the number of LAH present relative to AEH.

As barrier depth increased, the relative number of AEH and LAH per sample declined. Treatments at the ends of our experimental range (control and 50 cm) were always statistically significant. However, treatment groups one step apart (i.e. Control and 5 cm or 15 and 30 cm) were rarely statistically different. The rate of decline was influenced by seed volume, with the largest-sized grain, maize, showing the smallest reduction in the number of holes (AEH and LAH) relative to the control group (30% and 24%, respectively). Sorghum had the greatest reduction in AEH (52%), while sesame showed the greatest decline for LAH (58%). Multivariate regression analysis found no significant interaction effects between grain depth and seed volume for AEH.

Two-way ANOVA determined that dry grain mass increased with increasing barrier depth (Fig. 5c). A dry sample of 100 cowpeas weighs approximately 20 g. Control group samples weighed between 15.47 g (sorghum) and 17.58 g (sesame), while cowpea stored beneath 50 cm barriers weighed between 17.98 g (sorghum) and 19.09 g (sesame). This corresponds to a 5–14% increase in grain mass. However, seed depth was the only significant factor that influenced these observations and seed volume was not a relevant, explanatory factor.

## 4 Discussion

Earlier research has observed an inverse relationship between grain depth and the severity of damage inflicted on stored grain by insect pests (Simwat and Chahal, 1980, Martin et al., 2015). Our study confirms that as the distance between the site of an insect infestation and the outside air increases, there is a proportional decrease in the number of AEH observed in our 10 cm pipes. Shallow barriers of grain (5 cm), for example, did not significantly reduce the number of emerging adults relative to our control groups for both maize and sesame. But as the depth of these barriers increased from 15 to 50 cm, we observed the number of AEH decline proportionally.

Average seed volume also influenced AEH numbers, but there was not a clear relationship between these two variables. Maize and sesame (50 cm) barriers both had a 30–32% reduction in AEH counts (relative to our controls), but sorghum had the highest reduction at 50 percent. Because sorghum is intermediate in size relative to our other two grain types, we speculate that this unexpected result is an artifact of the high, initial infestation rate of the sorghum trial. The larger number of insects present in the sorghum control group may have caused the grain barrier to appear more effective, as we would naturally see more signs of damage in the control group pipes. Without this higher infestation level, we may have observed reductions in AEH number closer to what we observed in our maize and sesame trials. Excluding sorghum from our comparison revealed that while average seed volume remained a significant factor, its effect was very small and likely did not contribute much to the number of AEH observed.

Other signs of damage, such as LAH numbers, were also affected by grain depth. Additionally, there was a clearer relationship between LAH counts and average seed volume than between AEH and seed volume. Barriers made of the smaller-sized sesame (4 mm^3^) caused up to 58% reduction in LAH values. Meanwhile, the large-sized maize (170 mm^3^) reduced the number of holes by only a 24%. Sorghum (54 mm^3^) fell somewhere in between with a 43% reduction in the number of LAH.

How our grain barriers reduce insect damage and why they influence some aspects of insect damage to stored grain (LAH) more than others (AEH, seed weight) requires us to consider two factors: (1) the oxygen requirements of developing bruchids and (2) the movement of gases through the bulk grain. The combination of these two factors determines the amount of environmental stress experienced by the bruchid population in our infested 10 cm pipes.

Oxygen needs of our bruchid populations likely changed during the 72 d trial. We estimate that the first-generation populations within the 10 cm pipes ranged in number between 123 and 216 insects. This estimate is based on the average number of cowpeas (435) in each 10 cm pipe and infestation rate reported for our trials. Larval bruchids will consume 8.5 mL of oxygen over their developmental cycle (Murdock et al., 2012). This means that these first-generation populations would need between 75 and 131 mL of oxygen per day to develop normally.

The 10 cm pipes are too small to contain the oxygen needed for development, even for a day. Assuming that 39% of the volume in these pipes is air (Kabas et al., 2007, Davies and Zibokere, 2011) and that 20% of that air is oxygen, we estimate that the 10 cm pipes could only contain about 9 mL of oxygen. This volume is only about 7–12% of the populations’ estimated daily needs and is therefore insufficient for development (Murdock et al., 2012). Even if the insects adjust their respiration rates to use less oxygen (Murdock et al., 2012, Oxley and Wickenden, 1963), these populations still require oxygen from outside the pipe in order to survive.

These constraints only become more severe as the bruchid larvae develop towards adulthood. Under normal conditions, a female bruchid can lay ∼40 eggs during her lifetime. Assuming that half of the emerging adults are female and that only half of each female’s eggs hatch, we would still see a rapid increase in population size; from 120 to 216 beetles to 1200–2160. This higher number of insects requires larger volumes of air to sustain itself. Instead of a few hundred milliliters per day, these second-generation populations require 1.5–2.5 L of oxygen each day.

Moving such large volumes of oxygen through grain via diffusion becomes increasingly difficult as grain depth increases. A number of studies have looked at the movement of gases through grain, most often using maize, which we can use as a model for the other barrier grains. Previous research focused primarily on carbon dioxide (44.01 g/mol) diffusion through grain, but we can use this data to approximate the oxygen (32 g/mol) diffusion rates as both molecules are similar in mass. (Singh et al., 1984, Shunmugam et al., 2005, Haung et al., 2013, Haugh and Isaacs, 1967). We use the published data and the equation

(1) $$t=x^{2}/2D$$

to estimate how quickly carbon dioxide and oxygen move through our barriers. The variable *t* represents the elapsed time since diffusion began, *x* is the depth of our grain barrier, and *D* is the diffusion coefficient, Singh et al. (1984) calculated the diffusion coefficient of carbon dioxide through maize as 3.02 mm^2^/s, while Haugh and Isaacs (1967) estimated a slightly faster rate at 5.77 mm^2^/s. Using Graham’s Law and the molecular masses of the two gases, we estimate that oxygen diffuses through the grain about 17% faster than carbon dioxide (range 3.53–6.75 mm^2^/s).

Using these diffusion coefficients, we can estimate that a molecule of carbon dioxide or oxygen could pass through our 5 cm barrier of maize in about 3–6 min. Such a short amount of time to diffuse would not place significant stress on our bruchid population, especially for the first generation. However, the time required for both gases to pass through the grain barrier increases as the square of the length of the barrier. Instead of a few minutes, it would take a molecule of oxygen or carbon dioxide 5–10 h to pass through our 50 cm barrier of maize.

For the first generation of bruchids, this amount of time for the gases to move through the barrier would not be a significant constraint, but the greater needs of the second generation creates greater pressure on the system. Based on estimates provided by Bailey (1959), it would only take about an hour for the 150–260 mL of carbon dioxide produced by the first generation of bruchids to pass through the 50 cm maize barrier. However, the second generation produces more carbon dioxide and needs more oxygen. Moving this volume of gas in and out of the 10 cm pipes could take more than a day, during which, more oxygen is consumed and carbon dioxide is produced. The local environment would build a high concentration of carbon dioxide within and just above the 10 cm pipes. The result is a gradient of carbon dioxide and oxygen within the grain barrier. Even as carbon dioxide concentrations increase, the movement of the gas through the grain is independent of the gas’s concentration (Bird et al., 1960, Pritchard and Currie, 1982, Shunmugam et al., 2005), creating a positive feedback loop. This high CO2/low O2 balance would result in slower larval development within our 10 cm pipes, if not leading to the deaths of these insects during development. The effect would be greater on our second generation population than the first, due to their greater oxygen requirements. Many of the AEHs are likely the product of the first generation of bruchids. Meanwhile, few beetle larval in the second generation succeeded in reaching maturity and likely produced the greater number of LAH versus adult emergence holes observed in our trials.

Other factors such as temperature and grain moisture (Bailey 1959, Singh et al., 1984, Shunmugam et al., 2005, Haung et al., 2013, Haugh and Isaacs, 1967) play a role in this complex system. Changes in temperature have been observed to make insects respire faster or slower in response (Cofie-Agblor et al., 1996; Neven, 2000). This would make restrictions in oxygen diffusion by the grain more or less intense, as the insects’ metabolic rates change accordingly. Higher moisture would also decrease the severity of low-oxygen as the insects would be less prone to drying out (Navarro, 1978; Murdock et al., 2012; Murdock and Bauoa, 2014). For our study, though, we limited the influence of temperature and moisture from our trials to narrow the focus on just the effect the grain barrier’s depth and composition had on the system.

Another factor to consider is respiration by the barrier grains. We note that grain respiration consumes and produces a negligible amount of oxygen and carbon dioxide (on the order of μg/mL/day), respectively. This process is significantly less than what we estimated is produced by the insects (Table 4). As the amount of carbon dioxide produced by our barrier grains is less than 1% of that produced by our insects, we can rule it out as an influencing factor for our system.

The fact that our maize barrier was the lowest-performing barrier for suppressing pest damage of our three barrier grains confirms the importance of grain size/shape in reducing gas diffusion. The studies cited above pointed out that greater grain size (i.e., volume) leads to a more porous bulk density, which allows gases to flow more freely. This may aid in understanding how PICS bags work, and may assist in identifying which crops are more vulnerable to damage when the liner is compromised. Commodities that allow larger airspaces and greater freedom for gases to flow, like cassava chips (Hell et al., 2014) may be more vulnerable after bag damage than ones with higher resistance to gas flow, like sesame and sorghum.

## Figures and Tables

**Table 1:** Initial seed infestation for all three trials. Infestation rates are given as the percentage of seeds with at least 1 bruchid egg on its surface out of a 100 seed sample.

Trial	Mean infestation (%)	SE
Trial 1	39.75	1.43
Trial 2	50.25	3.42
Trial 3	28.25	1.75

**Table 2:** Estimated average volumes for kernels of three stored grains.

Grain	Avg. volume (mm^3^)	Source
Sesame	4.0	Tunde-Akintunde and Akintunder, 2004
Sorghum	54.0	Mwithiga and Sifuna, 2006
Maize	170.0	Tarighi et al. 2011

**Table 3:** Results of Two-way ANOVA comparing the effect of grain depth and seed volume on seed damage.

	Treatment	DF	F-value	P-value	R-square
Emergence Holes	Depth (D)	4	19.90	<0.001	68.53
	Volume (V)	2	83.25	<0.001	
	Error	113			
Air holes	Depth (D)	4	22.51	<0.001	71.60
	Volume (V)	2	97.46	<0.001	
	Error	113			
Seed Weight	Depth (D)	4	19.00	<0.001	56.84
	Volume (V)	2	36.41	<0.001	
	Error	113			

**Table 4:** Estimated daily CO2 production of barrier grains by volume and amount produced relative to carbon dioxide respired by *C. maculatus* populations in 10 cm pipes.

Depth (cm)	0	5	15	30	50
**Sesame**					
mL/day	NA	0.003	0.01	0.02	0.03
Grain/insects (%)	NA	(0.001, 0.003)	(0.004, 0.008)	(0.01, 0.013)	(0.012, 0.025)
**Sorghum**					
mL/day	NA	0.08	0.26	0.51	0.85
Grain/insects (%)	NA	(0.031, 0.053)	(0.1, 0.17)	(0.2, 0.34)	(0.33, 0.57)
**Maize**					
mL/day	NA	0.02	0.07	0.12	0.2
Grain/insects (%)	NA	(0.01, 0.013)	(0.027, 0.047)	(0.046, 0.08)	(0.077, 0.13)
